# American Dental Association

**Published:** 1874-09

**Authors:** 


### Proceedings of Societies.

### AMERICAN DENTAL ASSOCIATION.

The fouiteenth annual meeting of the American Dental Association began its session at St. Andrew's hall, Tuesday morning, Aug. 4th, 1874, and was called to order by the President, T. L. Buckingham, of Philadelphia. The meeting was opened with prayer by Rev. L. P. Mercer..

The roll being called. The following delegates and members were reported present:

F. H. Rehwinkle, C. R. Taft, Geo. W. Keely, C. R. Butler, John Stephan, A. H. Smith. J. Taft, E. J. Waye, H. L. Ambler, E. Osmond, Wm Taft, W. P. Norton, Geo. Watt, all of Ohio.

T. L. Buckingham, M. H. Webb, E. M. Wolfe, J. H. McQuillan, Geo. Elliot, C. D. Elliot, C. C. Carroll, V. M. McCall, O. S. Welchens, all of Pennsylvania.

Geo. H. Cushing, W. C. Dyke, J. S. Swartley, J. A. Swasey, E. D. Swain, M. S. Dean, J. N. Crouse, W. W. Allport, C. S. Smith, of Illinois.

W. H. Morgan, of Tennessee.

E. S. Gaylord, of Connecticut.

Jas. Johnson, of Virginia.

C. S. Stockton, of New Jersey.

S. E. Knowles, of California.

J. R. Walker and J. S. Knapp, of Louisiana.

Homer Judd, J. M. Austin, I. Forbes, of Missouri.

Geo. L. Field, G. R. Thomas, B. T. Spelman, D. C. Hauxhurst, of Michigan.

W. H. Atkinson, Jno. Allen, M. L. Chaim, E. A. Bogue, Corydon Palmer, W. A. Bronson, C. D. Cook, C. H. Biddle, A. N. Brockway, S. B. Palmer, S. Southworth, G. C. Dayboll, of New York.

W. H. Goddard and C. E. Dunn, of Kentucky.

L. D. Shepard, I. J. Wetherbee, of Massachusetts.

O. Kulp, of Iowa.

The first business taken up was amendments to the constitution. Sectiou 3, of article III, of the constitution was amended so as to elect honorary members by ballot.

An amendment to section 3 of article II of the code of dental ethics was proposed and adopted. It was as follows:

"It is unprofessional to resort to public advertisements, such as cards, hand bills, posters or signs calling attention to peculiar styles of work, prices for services, special modes of operating or to claim superiority over neighboring practitioners; to publish reports of cases, or certificates in the public prints; to go from house to house soliciting or performing operations; to circulate or recommend nostrums, or to perform any other similar acts. But nothing in this section shall be so construed as to imply that it is unprofessional for dentists to announce in :he public prints, or by card, simply their names, occupation and place of business; or, in same manner, to announce their removal, absence from or return to business; or to issue, to their patients, appointment cards having a fee-bill for professional services thereon."

ORDER OF BUSINESS.

Dr. Field of the Executive Committee reported the following order of business: Morning sessions from nine to halfpast twelve; afternoon sessions from half-past two to half-past five; evening session from half-past seven to adjournment; that Thursday afternoon be devoted to clinics. He also announced that the Detroit dentists invite the Association to take a boat ride on Wednesday afternoon, and ask that the Association give up the afternoon session of Wednesday for that purpose. The report was adopted except as to devoting Thursday afternoon to clinics, and this matter was laid on the table, and the invitation to the boat ride was accepted.

Dr. Shepard, of Boston, Mass., moved that all dentists and physicians resident in Detroit be invited to attend the meetings of the association. Carried.

The Association then adjourned until half-past two p. m.

### AFTERNOON SESSION.

The Association was called to order at half-past two o'clock by the President.

Dr. Dean, of Chicago, offered a resolution that no person shall be received as a delegate to the association who is in arrears for dues for previous membership. Adopted.

The Committee on Credentials made a supplementary report, and the following additional delegates were reported:

J. F. Canine, B. Oscar Doyle, Louisville, Ky.; E. Hunter, Manchester, Mich.; T. G. Noel, J. C Ross, Nashville, Tenn.; H. K. Lathrop, Jr., James Cleland, Detroit; A. T. Metcalf, Kalamazoo; Wm. Van Antwerp, Mt. Sterling, Ky.; S. B. Tizzard, Dayton, Ohio; I. Douglass, Romeo, Mich.; A. J. Grosvenor, Troy, O.

The Committee on Physiology, through Dr. Dean, of Chicago, presented a report upon the absorption of the roots of deciduous teeth. It was a scientific paper, and was characterized by research and ability. Prof. J. H. McQuillen, at the request of the convention, also read a paper on the same subject, which was published in the Dental Cosmos, 1860, wherein he accounted for absorption of the roots of deciduous teeth by attributing it to the operation of the law of waste and supply governing the entire economy by which atrophy, or wasting of soft tissues takes place through retrograde metamorphosis, or degeneration of the cells entering in to their composition.

Dr. Atkinson, of New York, then spoke upon the question at length, setting forth the manner in which such absorption takes place. Dr. Judd, of St. Louis, was also heard from, and the further consideration of the matter was postponed until the evening session.

Dr. Crouse, of Chicago, from the Committee on the Testimonial to Dr. Barnum, of New York, the inventor of the rubber dam, reported progress in the collection of funds, and stated that efforts would be made at the present session to raise the thousand dollars required far this purpose.

The Association then adjourned until 7:30 p. m.

EVENING SESSION.

The Association was called to order at eight o'clock p. m. the President in the chair.

The subject under consideration during the afternoon was taken up and its discussion resumed by Dr. Atkinson, of New York; Dr. Judd, of St. Louis; Dr. Dean, of Chicago; Dr. J. Taft, of Cincinnati; and Dr. Knapp, of New Orleans.

Dr. Geo. A. Cushing, of Chicago, from the Committee on Pathology and Surgery, read a report on the subject of dental pathology, prepared by Dr. H. S. Chase, of St. Louis.

The paper was discussed at some length by Dr. Atkinson, of New York; Dr. Judd, of St. Louis; Dr. Bogue, of New York; Dr. Charles Butler, of Cleveland; Dr. W. H. Morgan, of Nashville; and J. R. Walker, of New Orleans.

Dr. Bogue in the course of his remarks spoke of the toothache as being inflammation of the pulp, or what is often called the nerve of the tooth. In some cases he has stopped the aching almost immediately by inserting a sharp instrument into the pulp and letting out a small portion of the blood gathered there. In the course of the discussion the question arose as to whether there is any sensation in the enamel, it having been brought up by the assertion in Dr. Chase's paper that there was a circulation going on in the enamel. It was the opinion of some that there could be no circulatiun, but that enamel was subject to permeating influences necessary to its support and healthy condition, and that the sensation caused by acids of "setting teeth on edge" was only felt when there was some defect in the enamel.

The Association at ten o'clock adjourned to meet at the common council chamber at half-past nine Wednesday morning.

### 2d DAY, MORNING SESSION.

The Association assembled Wednesday morning at nine o'clock, at the common council chamber, the President, Dr. T. L. Buckingham in the chair.

The subject of Dental Pathology was resumed, and discussed by Drs. Taft, Spelman, Geo. Watt, W. H. Morgan, Homer Judd, W. H, Atkinson, M. S. Dean and E. A. Bogue.

The subject was then passed and the Committee on Histology called upon for a report, but asked for further time which was granted.

The following committee was appointed to draft resolutions on the death of Dr. T. B. Hitchcock, of Harvard Dental College, Drs. Bogue, J. Taft and Shepherd.

Dr. H. A. Smith, of Cincinnati, Chairman of the Committee on Dental Chemistry, read a paper on that topic.

Dr. Shepherd, of Boston, offered an amendment to the constitution, giving the Executive Committee power, for any extraordinary reasons, to change the time and place of holding the meetings of the Association which, under the rules, was laid over.

On motion of Dr. J. Taft, of Cincinnati, clinics were dispensed with during this session.

The Association then adjourned to 7:30o'clock this evening.

During the afternoon the members of the association enjoyed the very pleasant excursion and entertainment given by the dentists of Detroit, for which they received the most hearty thanks of the Association.

### 2d DAY, EVENING SESSION,

The association met at eight o'clock, and the Committee on Histology not being ready to report, were granted further time.

THERAPEUTICS.

Dr. Taft moved the appoitment of a committee to offer some remarks on the subject of therapeutics. The motion prevailed and the chair appointed as such committee Dr. Taft.

ETIOLOGY.

Dr. Webb, of Lancaster, Pa., from the Committee on Etiology, read a paper upon etiology or the "Science of Causes." It was a resume of researches into the various causes of decay of the teeth, with the manner of treatment of various cases.

The paper was referred to the Committee on Publication.

Dr. McQuillan, also a member of same committee, was called upon for an additional report, but stated he had only an oral report to make, and to make this he must have a black board to illustrate the topic by drawings to represent microscopic plates. The further consideration of the subject was deferred until this morning.

OPERATIVE DENTISTRY.

Dr. Shepard, of Boston, then read a paper upon the subject of operative dentistry. He said that various machines for operating upon the teeth had been invented and brought to use during the past few years, and that more machinery would doubtless be brought into use within the next few years. To the treadle machines he said there were objections. Electric machines had been tried but without entire satisfaction. There were great objections to the use of steam as a motor. Water had been used as a motor, but this also had objections. He had seen a spring motor which, when wound up, would run a sewing machine for twenty minutes, and this machine met with more favor from him than any other. Heavy mallets were brought into use a few years since, which it has been thought were better for welding gold. He then mentioned various instruments for filling teeth, and for doing other work connected therewith lately invented and brought into use among dentists. He said that platinum had been used in place of gold, and when used with gold for welding purposes makes a good filling. He also mentioned an amalgam which had proved excellent. It is made of tin 130 parts, silver 30 parts and mercury 25 parts.

The greatest improvements of late years have been cohesive gold and the rubber dam, and all honor is due to Robert Arthur and S. C. Barnum, their respective discoverers. No one can now do the best work without these two improvements. For the past four or five years he has been impressed with the question, what can he do to preserve the teeth of his patients? The filling of teeth to-day is a longer process than it was several years ago. It may also be said that it is a more painful one, yet this is needed in order to preserve the teeth. Every moment spent upon a tooth more than necessary is misspent time. The paper was a very interesting one.

The subject was then discussed by Drs. Southworth, of Niagara Falls; Crouse, of Chicago; Wetherbee, of Boston; and Taft, of Cincinnati.

Dr. Taft announced that as many had expressed great desire to witness some operations, arrangements had been made for holding clinics at St. Andrew's hall at eight o'clock to-morrow morning, and that Drs. Butler, Webb and Ambler would operate.

The Association then adjourned.

### 3d DAY, MORNING SESSION.

The Association assembled at ten o'clock, Thursday, the President, Dr. T. L. Buckingham in the chair.

Dr. F. H. Rehwinkle, of Chillicothe, O,, from the committee appointed to confer with the Surgeon General of the army of the United States for the appointment of dental sugeons for the army, reported that with the tendency of Congress toward cutting down instead of increasing the army, the committee deemed it inexpedient at the present time to make any such request. The report was adopted and the committee discharged.

The subject of operative dentistry was resumed and Dr. Cushing read a paper upon heavy *vs.* light foil for filling teeth, expressing a preference for heavy foil and giving it as his opinion that heavy foil, in the hands of a skillful operator, will produce the best results.

Dr. Butler, of Cleveland, doubted whether, as was asserted in the paper read last evening by Dr. Shepard, there was more time spent in gold fillings than there was fifteen or twenty years ago. If there is more time spent it is because dentists now undertake more difficult operations, and a finer finish is demanded than formerly by patients; a fine piece of workmanship takes time in its accomplishment. He advocated the use of the rubber dam in almost every case of filling. With reference to the kind of gold used, he thought it depended upon the work to be done, and the operator must judge where each kind should be used.

Dr. Knapp, of New Orleans, commended very highly the report of Dr. Shepard on operative dentistry. He differed with him, however, in giving the credit to Dr. Arthur for the preparation of adhesive gold, and said that the older members of the profession will remember that Dr. Leach, of Baltimore, used gold fully as adhesive as any ever used by Dr. Arthur, fully thirty years ago. The assertion made by Dr. Shepard that only the best work can be accomplished by the rubber dam and adhesive gold be considered a fallacy, and stated that the best of work has been done without the use of either. There are cases where the rubber dam should be used, but its use is generally carried to an extreme. He also believed the use of machinery was carried to a too great an extent, and that, in certain cases, where the decay extends close to the pulp, a devitalized pulp results from the use of machinery.

Dr. John Allen thought it mattered but little what kind of gold was used, or what method was employed, so long as good work was done. He can call to mind many fillings put into teeth forty years ago that are doing service now. He believed that taking the mass of dentistry to-day its work is not so good as it was thirty years ago.

Dr. Keely, of Oxford, O., believed that with heavy gold, the burring engine, the mallet and rubber dam the best results can be secured.

Dr. Kulp, of Davenport, Iowa, said that during the past year he has examined the fillings placed in different people's mouth's by twenty-two different operatars present at at this Association, the patients coming from the East. The results of this examination led him to believe that those who have followed the modern improvements have put in the best fillings. He did not believe in vacillating in the use of different kinds of gold, and thinks that one kind steadily used is the best. He uses No. 4 gold, and considers it the best for general use.

Dr. Rehwinkle, of Chillicothe, said we had to battle with the abuse of good things. The judicious use of the rubber dam he regarded as of great importance, but there are some young dentists who can not do any work except by the use of the rubber dam and adhesive gold. No preceptor should allow a student to go out of his office unless he was able to fill a tooth without the use of the rubber dam, and with non-cohesive gold. The condition of the tooth and the health of the patient he thought had much to do with the result in most cases.

Dr. Stockton, of New Jersey, thought there were some kinds of teeth that could only be treated with soft gold, while others could only be treated with adhesive gold. He thought that the appliances of to-day were so good that every filling should be so thoroughly tight as to last a lifetime.

Dr. Osmond was of the opinion that other material could be used with as much success as gold, and that in certain conditions of the teeth it is not practicable to use the mallet upon them, as it will break them.

Dr. Judd, of St. Louis, said there was a demand for all kinds of dentistry, especially in large cities; that some dentists have a class of patients who demand the very best work finished in the highest style of art; there others who are not so particular, and so the demand runs down to the cheapest kind of work. Dr. Judd thought Dr. Shepard was too conservative in his remarks on the rubber dam, and spoke of it as one of the most important appliances of the profession. He was not ready to endorse tin as a filling, in place of gold, and said that a filling of soft gold could be made as easy as tin. He thought that in many cases the teeth were not cut out enough. He believed in filling the canals of teeth with gold, and was quite sure fangs should be filled with nothing but gold. He was satisfied that the canals of teeth could not always be entirely filled up with gold. With reference to operating, he said there were very many fine operators in the United States who were not good dentists, and there was more injury from injudicious filling of teeth than was necessary.

Dr. Shepard, of Boston, maintained that in certain cases tin-foil was best to be used. The majority of people, he said, are poor and they must be treated as skillfully as the best. If their teeth can be saved, the dentist who operates upon them and preserves them for $1 a cavity is as much a benefactor as the dentist who works for rich people and charges $10 per hour. The amount of gold required to fill a tooth costs to much to be afforded by poor people.

Prof. McQuillen, of Philadelphia, advocated the very best work under all circumstances, whether the patient be able to pay for the services or not.

Dr. Allport, of Chicago, thought the gold used should be that kind which the average dentists of the coun'ry can use to the best advantage, and he considered that soft gold in the hands of the average dentist saved more teeth than the adhesive gold because it can be more easily packed to the side of the tooth. He considered the rubber dam as a most important adjunct to the filling of teeth, and he used it invariably.

Dr. Bogue, from the committee appointed to draft resolutions relative to the death of Prof. Hitchcock, reported the following:

WHEREAS, This association has with sorrow learned of th death of Prof. Thos. B. Hitchcock, of the Harvard Dental School, one of our most valued members, and chairman for this year of the Committee on Histology and Microscopy therefore

*Resolved*, That we signify in this public manner our sense of great losss which the profession and the cause of dental education has sustained, and that this resolution be inserted in the records, at the place where the report of the committee would have appeared.

The association then adjourned to half-past two o'clock p. m.

